# Factors associated with 6‐min walk distance in severe asthma: A cross‐sectional study

**DOI:** 10.1111/resp.14323

**Published:** 2022-07-10

**Authors:** Anders Pitzner‐Fabricius, Vanessa L. Clark, Vibeke Backer, Peter G. Gibson, Vanessa M. McDonald

**Affiliations:** ^1^ Centre for Physical Activity Research Copenhagen University Hospital—Rigshospitalet Copenhagen Denmark; ^2^ Centre of Excellence in Severe Asthma and Priority Research Centre for Healthy Lungs Hunter Medical Research Institute, College of Health Medicine and Wellbeing, The University of Newcastle Newcastle New South Wales Australia; ^3^ Department of Otorhinolaryngology Rigshospitalet, University of Copenhagen Copenhagen Denmark; ^4^ Department of Respiratory and Sleep Medicine John Hunter Hospital Newcastle New South Wales Australia

**Keywords:** 6‐min walk test, BMI, exercise capacity, quality of life, severe asthma

## Abstract

**Background and objective:**

Exercise capacity is associated with health‐related quality of life and symptom control in severe asthma. Thus, interventions targeting exercise capacity are likely to be beneficial. However, clinical and biological factors impacting exercise capacity in severe asthma are sparsely investigated. We aimed to describe the association of selected clinical and biological factors with 6‐min walk distance (6MWD) in adults with severe asthma and investigate the impact of sex on these outcomes.

**Methods:**

A cross‐sectional study in adults with severe asthma was conducted. Exercise capacity was measured by 6‐min walk test, and association between 6MWD and predictors were evaluated using multiple linear regression.

**Results:**

A total of 137 patients (females, 85; median age, 59 years) were recruited. Overall, asthma control (−15.2 m, 95% CI −22.6 to −7.7; *p* = 0.0001) and BMI (−3.2 m, 95% CI −5.1 to −1.3; *p* = 0.001) were significantly associated with exercise capacity (adjusted variance, adj. *R* ^2^ = 0.425). In females, 5‐item Asthma Control Questionnaire (ACQ‐5; *p* = 0.005) and BMI (*p* < 0.001) were significantly associated with 6MWD (adj. *R* ^2^ = 0.423). In males, a 0.5‐point increase in ACQ‐5 was associated with a decrease in 6MWD by 10.2 m (95% CI −22.8 to 2.4; *p* = 0.11), but no clinical nor biological factors reached statistical significance (adj. *R* ^2^ = 0.393).

**Conclusion:**

Asthma symptoms and BMI were associated with exercise capacity in the overall population. Optimizing these factors may enhance the ability of patients to improve their exercise capacity and gain the associated positive health outcomes, but further studies are warranted.

## Abstract 2

Among adults with severe asthma in Australia, association between 6‐min walk distance (6MWD) and clinical and biological factors was examined. Asthma control and BMI were significantly associated with 6MWD.

**See related** Editorial

## INTRODUCTION

Severe asthma is a complex and heterogenous disease associated with high disease burden, impaired health‐related quality of life (HRQoL) and multimorbidity.^1^, ^2^ Given the complexity of the disease pathobiology and high prevalence of comorbidities impacting disease control, a multidimensional assessment approach has been proposed to identify treatable traits.^3^, ^4^, ^5^ In addition to pharmacological treatment, extra‐pulmonary traits and behavioural/risk factors including physical inactivity and deconditioning are recognized as important, as these are modifiable risk behaviours.^6^

Aerobic exercise improves many health outcomes, including risk of cardiovascular disease, cardiorespiratory function and mental health.^7^, ^8^ In severe asthma, physical activity level associates with HRQoL and asthma control.^9^, ^10^ Adults with severe asthma perform less moderate to vigorous physical activity and take fewer steps/day compared to healthy controls.^10^, ^11^, ^12^ Levels of physical activity in asthma are influenced by sex, with females, especially middle‐aged and older, engaging in less physical activity than males.^12^ With hormonal and structural sex differences, women have a pulmonary disadvantage when exercising compared to men.^13^ In the general population, BMI is more closely associated with exercise capacity in women than men.^14^ In asthma, disease severity has been associated with BMI in females, but not in males.^15^ Moreover, the adult‐onset obesity‐related asthma phenotype is generally associated with more symptoms and predominantly consists of females.^16^, ^17^ Consequently, it is plausible that interaction between factors such as asthma symptoms, exercise capacity and BMI differs between females and males.

Few studies have tested exercise interventions in people with severe asthma disease, and in those that have there are concerns about retention rate.^18^ Identifying and treating factors that impact exercise capacity should allow individuals to improve their exercise capacity and gain the associated health benefits. Therefore, elucidating the impact of clinical and biological factors on exercise capacity level is needed.

In this study, we aim to select clinical and biological factors with plausible impact on exercise capacity. Moreover, we want to describe the associations of these factors with 6‐min walk distance (6MWD) and investigate if they impact differently across sexes. We hypothesized that symptoms, BMI, systemic inflammation, anxiety, depression, hospitalization and cardiac disease were negatively associated with exercise capacity, while leg strength and lung function would be positively associated. In addition, we hypothesized that BMI would have a stronger negative impact on exercise capacity in women.

## METHODS

### Study participants

This is an analysis of a previously reported cross‐sectional study in adults with severe asthma.^3^ Patients with severe asthma were consecutively recruited from John Hunter Hospital (Newcastle, NSW, Australia). Adults (≥18 years) with a respiratory physician diagnosis of severe asthma were eligible. Severe asthma was based on the European Respiratory Society/American Thoracic Society (ERS/ATS) guidelines: treatment with 1000 mcg or greater of inhaled corticosteroid fluticasone equivalent and long‐acting β2‐agonists or maintenance oral corticosteroids; evidence of airflow limitation (forced expiratory volume in 1 s [FEV1] post β2‐agonist <80% predicted or FEV1/forced vital capacity [FVC] <70%); poor Asthma Control Questionnaire (ACQ) score ≥1.5 or had experienced a severe exacerbation in the last 12 months requiring oral corticosteroids; current or prior demonstration of variable airflow limitation (reversibility to bronchodilator of 12% or 200 ml, airway hyperresponsiveness or diurnal peak flow variation ≥15%).^19^ Participants had stable asthma symptoms without any exacerbation 4 weeks prior to the study visit. Individuals were excluded if they were diagnosed with malignancy or had a poor prognosis with less than 3 months of life expectancy.

### Procedures

#### Clinical measurements

Participants underwent a multidimensional assessment, involving measurement of height and weight and medical history including self‐reported history of cardiac disease, anxiety and depression.^4^ Exacerbations in the previous 12 months were registered. An exacerbation was defined as worsening of respiratory symptoms that required treatment with oral corticosteroids or antibiotics.

#### Exercise capacity

The 6‐min walk test (6MWT) was performed according to the ERS/ATS guidelines.^20^, ^21^ The 6MWD was the reported outcome. Percent of expected normal value of 6MWD was calculated.^14^ Self‐reported limitations to the 6MWT was recorded (Appendix S1 in the Supporting Information).

#### Isometric strength test

Isometric leg strength was assessed using a lower limb and back platform dynamometer (Baseline®, USA). Two tests were performed in both legs. The highest reading on the strongest leg was included in multiple regression analysis. Values from participants who were unable to hold the leg test position (e.g., due to musculoskeletal pain) were excluded from the analysis.

#### Lung function

Pre and post FEV1, FVC and FEV1/FVC ratio was assessed by measuring spirometry (Medgraphics, CPFS/D USB Spirometer; BreezeSuite v7.1, MGC Diagnostics, Saint Paul, MN).^22^ Equations from NHANES III were used to calculate predicted values.^23^ Spirometry was conducted on a different day to the 6MWT.

#### Systemic inflammation

Systemic inflammation was determined by peripheral blood IL‐6, measured in duplicate by immunoassay via commercially available ELISA kits as per manufacturer's instructions.

#### Asthma control

Asthma control was assessed with the 5‐item Asthma Control Questionnaire (ACQ‐5).^24^ The measure consists of five questions regarding asthma symptoms and activity limitations. Each question scores 0–6 points, with a higher score interpreted as worse asthma control.^25^ A change of 0.5 is considered the minimal clinically important difference.

#### Health status

Health status was measured using the Juniper Asthma Quality of Life questionnaire (Appendix S1 in the Supporting Information).^26^

### Statistical analysis

Data were analysed using SPSS statistics 25 (IBM, Chicago, USA). Values are expressed as means with 95% CI for parametric data and medians with quartiles 1 and 3 (Q1 and Q3) for non‐parametric data. Differences between sex were assessed using the independent Student's *t*‐test and Mann–Whitney *U*‐test based on normality, or Pearson's chi square or Fischer's exact test. Age was regarded as a biological confounder and included in all models. Analyses were performed in the overall population and split by sex. Split was performed as the interaction between predictors, both biological and asthma‐related, and exercise capacity was likely to differ between sexes. The association between the 6MWD and each clinical and biological variable was estimated using simple linear regression (SLR). Each clinical and biological variable (independent variables: ACQ‐5, FEV1 % predicted, BMI, hospitalization due to exacerbation prior 12 months [yes/no], anxiety, depression, cardiac disease [coronary heart disease, arrhythmia, heart failure or pericarditis], isometric leg strength and IL‐6) were used as a predictor of the 6MWD. Independent variables with a *p* value of less than 0.20 were included in a multiple linear regression (MLR) analysis to estimate how well our combined clinical and biological variables predict the level of 6MWD. We also performed a sensitivity analysis, where all independent variables were included in the MLR model. Categorical predictors were assigned a dummy value (0 = ‘no’ or male; 1 = ‘yes’ or female). Results in the regression analyses are presented as mean change in walking distance measured in metre (m) per 1‐unit (0.5 for ACQ‐5) change in the biological or clinical factors and/or a correlation coefficient (*β*). Assumptions for linear regressions were met. Multicollinearity was ruled out by variance inflation factor. Results are reported as significant when *p* <0.05.

## RESULTS

### Characteristics of study population

**FIGURE 1:**
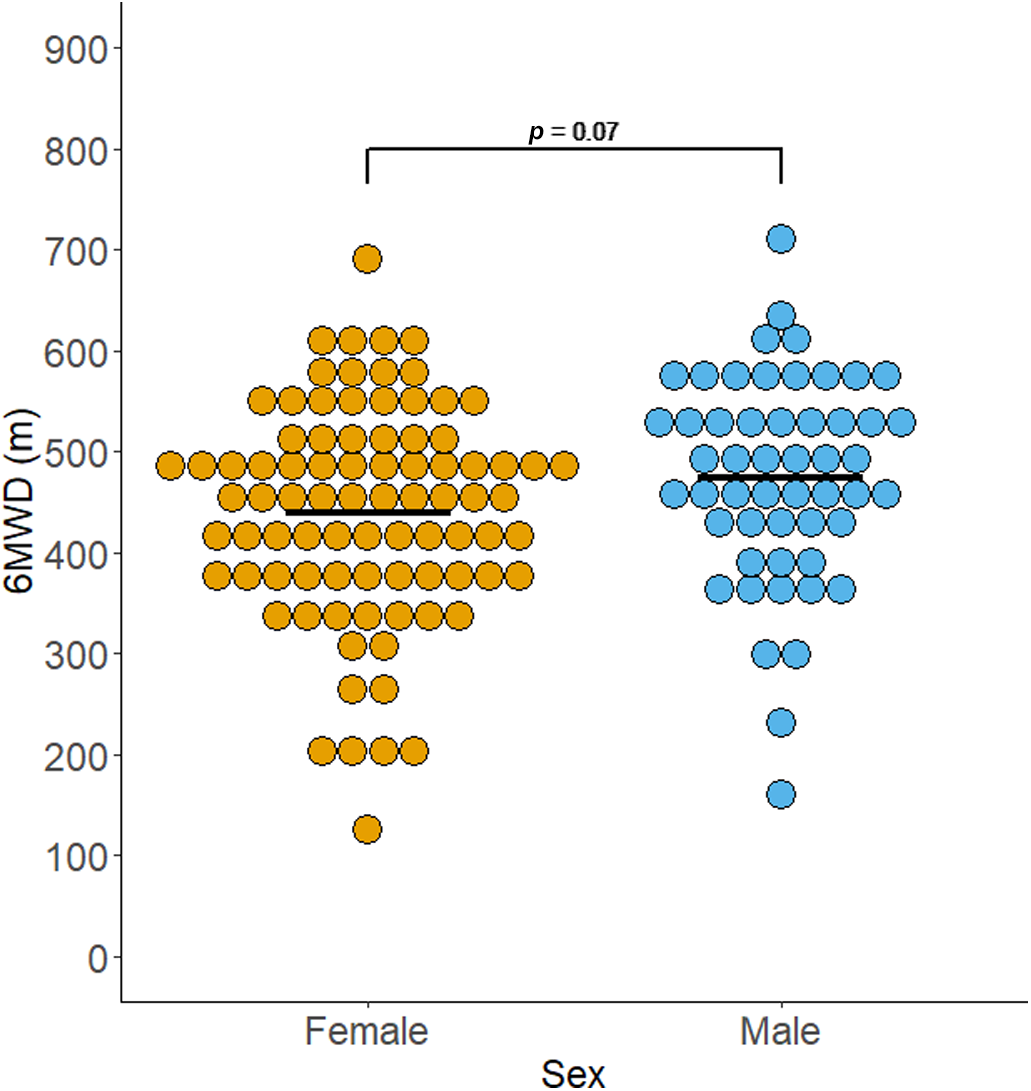
Comparison of 6‐min walk distance (6MWD) in males and females. Unadjusted dot‐plot of 6MWD split by sex. Solid line indicates mean.

A total of 137 participants (females, 85 [63.5%]; median age, 59 years) with severe asthma were included in this analysis. No differences were seen between sexes in most clinical and demographic variables (Table 1). Females had worse HRQoL (*p* = 0.001) and a tendency to have more asthma symptoms (*p* = 0.051), while males had greater leg strength. Males had a mean 6MWD of 473 m compared to 440 m in females (mean difference of 34 m [95% CI −3 to 71; *p* = 0.07]; Figure 1). Adjusted for age, females walked 45 m less (95% CI −11 to −79; *p* = 0.01) than males.

**TABLE 1:** Demographics and comparison between sex

	Total	Male	Female	Difference between sexes: *p* value
	*n* = 137	*n* = 52	*n* = 85	
Age (years), median (Q1–Q3)	59 (44–68)	61 (47–69)	57 (43–66)	0.14
BMI (kg/m^2^)	31.7 (30.3–33.1)	31.0 (29.4–32.6)	32.1 (30.1–34.1)	0.37
Current smoker	6.6 (9)	1.9 (1)	9.4 (8)	0.15
Ex‐smoker	38.2 (64)	50.0 (26)	44.7 (38)	0.55
Pack‐years, geometric mean	5.5 (3.7–8.2)	4.8 (2.4–9.8)	6.1 (3.8–9.7)	0.56
Year since diagnosis	33.8 (30.3–37.2)	34.5 (28.7–40.3)	33.3 (28.9–47.7)	0.74
Oral corticosteroid (daily)	27.0 (37)	34.6 (18)	22.4 (19)	0.12
Inhaled corticosteroid dose^a^ (μg)	1733 (1532–1933)	1631 (1523–1738)	1795 (1477–2113)	0.43
Prebronchodilator FEV1 (L)	2.15 (2.01–2.27)	2.38 (2.13–2.63)	2.00 (1.86–2.15)	NA
Prebronchodilator FEV1 % predicted	73.7 (70.0–77.3)	70.1 (64.0–76.3)	75.8 (71.2–80.3)	0.14
Prebronchodilator FVC (L)	3.19 (3.03–3.34)	3.71 (3.42–4.00)	2.87 (2.71–3.02)	NA
Prebronchodilator FVC % predicted	84.8 (82.0–87.6)	83.5 (78.4–88.6)	85.6 (82.3–88.9)	0.48
FEV1/FVC ratio	0.67 (0.65–0.69)	0.63 (0.60–0.67)	0.69 (0.66–0.72)	**0.014**
FeNO ppb, geometric mean	15 (12–19)	16 (12–21)	15 (11–20)	0.75
Sputum eosinophilia (%), median (Q1–Q3)	3.5 (0.8–12.7)	3.3 (0.6–12.9)	4.0 (0.8–12.8)	0.85
Sputum neutrophils (%), median (Q1–Q3)	39.8 (18.1–61.6)	41.3 (18.1–61.1)	34.9 (17.3–61.8)	0.76
IL‐6 (pg/ml), median (Q1–Q3)	2.32 (0.92–4.10)	2.37 (1.05–3.34)	2.26 (0.74–4.39)	0.85
Anxiety	20.4 (28)	15.4 (8)	23.5 (20)	0.25
Depression	38.0 (52)	23.1 (12)	47.1 (40)	**0.005**
Cardiac disease^b^	27.7 (38)	34.6 (18)	23.5 (20)	0.16
Hospitalization^c^	24.1 (33)	19.2 (10)	27.1 (23)	0.30
AQLQ	4.9 (4.7–5.1)	5.3 (5.0–5.6)	4.6 (4.4–4.9)	**0.001**
ACQ‐5	2.0 (1.9–2.2)	1.8 (1.5–2.1)	2.2 (2.0–2.4)	0.051
Best leg, isometric strength (kg m/s^2^)	78 (70–87)	117 (102–131)	55 (47–62)	**<0.0001**
6MWD m walked	452 (434–470)	473 (445–502)	440 (417–463)	0.07
6MWD % predicted^d^	69.9 (67.5–72.3)	66.4 (62.6–70.1)	72.0 (69.9–75.1)	NA

*Note*: Data are presented as mean (95% CI) or percentage (numbers) unless otherwise stated. Bold *p* value denotes statistical significance (<0.05).

Abbreviations: 6MWD, 6‐min walk distance; ACQ‐5, 5‐item Asthma Control Questionnaire; AQLQ, Asthma Quality of Life Questionnaire; FeNO, fractional exhaled nitric oxide; FEV1, forced expiratory volume in 1 s; FVC, forced vital capacity; NA, not applicable or not assessed; Q, quartile.

^a^ Inhaled corticosteroid dose in budesonide equivalent.

^b^ Cardiac disease: coronary heart disease, heart failure, arrhythmia and pericarditis.

^c^ Asthma exacerbation resulting in hospitalization.

^d^ Equation with adjustment for sex and age in both sexes, and BMI in females.

### Associations of exercise capacity with clinical outcomes and comorbidities

Results from the SLR analyses are presented in Table 2. Using MLR in the overall group, an increase of 0.5 points in asthma control and 1‐unit increase in BMI were significantly associated with a change in exercise capacity by −15.6 m (95% CI −22.6 to −7.7; *p* = 0.0001) and −3.2 (95% CI −5.1 to −1.3; *p* = 0.001), respectively (Table S1 and Appendix S1 in the Supporting Information). Sex, leg strength, IL‐6, FEV1 % predicted, hospitalization, anxiety and depression were not significantly associated (*p* > 0.05). The model explained 42.5% of the adjusted variance (adj. *R* ^2^) in 6MWD. When split by sex, asthma control and BMI remained significant in females (adj. *R* ^2^ = 0.423). In males, only age was significantly associated with the 6MWD in the MLR model (adj. *R* ^2^ = 0.393) (Tables 3 and 4).

**TABLE 2:** Association of biological and clinical factors with 6MWD using a single linear regression model adjusted for age

	Simple linear regression model								
	Both sexes^a^			Males^b^			Females^b^		
6MWD	Mean change (95% CI)	*β*	Sign.	Mean change (95% CI)	*β*	Sign.	Mean change (95% CI)	*β*	Sign.
ACQ‐5	−18.7 (−25.7 to −11.6)	−0.395	**<0.0001**	−17.1 (−27.5 to −6.8)	−0.388	**0.002**	−20.4 (−30.4 to −10.4)	−0.412	**0.0001**
BMI	−4.4 (−6.3 to – 2.5)	−0.337	**<0.0001**	−2.5 (−7.1 to 2.1)	−0.139	0.29	−4.9 (−7.0 to −2.8)	−0.425	**0.0001**
Pre‐BD FEV1 % predicted	0.6 (−0.2 to 1.5)	0.123	0.12	0.6 (−0.7 to 1.8)	0.122	0.36	0.6 (−0.4 to 1.6)	0.124	0.22
Hospitalization	−40 (−79 to −2)	−0.162	**0.041**	−19 (−90 to 52)	−0.073	0.59	−51 (−98 to −3)	−0.212	**0.037**
IL‐6, pg/ml	−3.9 (−7.9 to 0.0)	−0.161	0.051	−0.9 (−10.3 to 8.6)	−0.027	0.85	−4.7 (−9.1 to −0.2)	−0.213	**0.039**
Leg strength	0.6 (0.2 to 0.9)	0.286	**0.001**	0.8 (0.2 to 1.5)	0.338	**0.015**	0.4 (−0.4 to 1.2)	0.122	0.327
Cardiac disease	−19 (−59 to 20)	0.081	0.33	−18 (−78 to 42)	−0.083	0.55	−20 (−74 to 33)	−0.080	0.46
Anxiety	−31.9 (−73 to 9)	−0.121	0.12	−56 (−130 to 17)	−0.199	0.13	−21 (−71 to 29)	−0.084	0.41
Depression	−46 (−81 to −12)	−0.211	**0.009**	−62 (−123 to 0)	−0.253	**0.048**	−39 (−82 to 3)	−0.184	0.07
Age	−2.7 (−3.8 to 0.1.6)	−0.400	**<0.0001**	−3.0 (−4.9 to −1.1)	−0.414	**0.002**	−2.8 (−4.2 to −1.4)	−0.395	0.**0002**
Sex	−45 (−79 to −11)	−0.206	**0.01**	NA	—	—	NA	—	—

*Note*: Results are presented as mean change in 6MWD (in metres) per 1‐unit change (0.5 for ACQ‐5) in the biological or clinical factor, and as a regression coefficient (*β*). Bold *p* value denotes statistical significance (<0.05).

Abbreviations: 6MWD, 6‐min walk distance; ACQ‐5, 5‐item Asthma Control Questionnaire; FEV1 (%), percent of predicted forced expiratory volume in 1 s; pre‐BD, prebronchodilator; sign., significance.

^a^ Adjusted for age and sex.

^b^ Adjusted for age.

**TABLE 3:** Association of biological and clinical factors with 6MWD in females using a MLR model adjusted for age

	MLR analysis in females			
6MWD	Mean change (95% CI)	*β*	Significance	Adj. *R* ^2^
ACQ‐5	−14.5 (−24.5 to −4.5)	−0.303	**0.005**	0.423
BMI	−4.0 (−6.0 to −1.9)	−0.352	**<0.001**	
Hospitalization	−20 (−63 to 24)	−0.082	0.365	
IL‐6, pg/ml	−3.2 (−7.0 to 0.7)	−0.144	0.104	
Depression	9 (−32 to 49)	0.041	0.668	
Age	−3.5 (−4.9 to −2.2)	−0.500	**<0.00001**	

*Note*: Results are presented as mean change in 6MWD (in metres) per 1‐unit change (0.5 for ACQ‐5) in the biological or clinical factor, and as a regression coefficient (*β*). Bold *p* value denotes statistical significance (<0.05).

Abbreviations: 6MWD, 6‐min walk distance; ACQ‐5, 5‐item Asthma Control Questionnaire; Adj. *R* ^2^, adjusted variance; MLR, Multiple linear regression.

**TABLE 4:** Association of biological and clinical factors with 6MWD in males using a MLR model adjusted for age

	MLR analysis in males			
6MWD	Mean change (95% CI)	*β*	Significance	Adj. *R* ^2^
ACQ‐5	−10.2 (−22.8 to 2.4)	−0.227	0.109	0.393
Isometric leg strength	0.6 (−0.1 to 1.2)	0.238	0.073	
Anxiety	−50 (−115 to 15)	−0.192	0.124	
Depression	−24 (−94 to 46)	−0.096	0.489	
Age	−2.9 (−4.8 to −1.1)	−0.403	0.**003**	

*Note*: Results are presented as change in 6MWD (in metres) per 1‐unit change (0.5 for ACQ‐5) in the biological or clinical factor, and as a regression coefficient (*β*). Bold *p* value denotes statistical significance (<0.05).

Abbreviations: 6MWD, 6‐min walk distance; ACQ‐5, 5‐item Asthma Control Questionnaire; MLR, multiple linear regression.

### BMI and IL‐6

With a mean BMI of 31.0 and 32.1 kg/m^2^ for males and females, respectively, no difference was seen between sexes (*p* = 0.37). In the MLR analysis, BMI was highly significantly correlated, with an inverse relationship, to 6MWD in females (*β* = −0.352, *p* < 0.001). A reduction of 1 unit in BMI was associated with an increase of 4.0 m (95% CI 1.9–6.0) in walk distance. BMI was non‐significant in the analyses in males. IL‐6 was inversely, but non‐significantly, associated with 6MWD in females, improving walk distance by 3.2 m (95% CI 0.7–7.0; *p* = 0.10) per reduction of 1 pg/ml in IL‐6 concentration (Table 3). Same tendency was observed in the overall population (*p* = 0.058).

### Leg strength and lung function

FEV1 % of predicted was non‐significant in all analyses. In the SLR analysis, leg strength was positively, and significantly, associated with 6MWD in the entire group and in males (0.8 m, 95% CI 0.2–1.5), with an increase of 1 newton associated to an improvement in walk distance of 0.6 m (95% CI 0.2–0.9; *p* = 0.001) in the overall group and 0.8 m (95% CI 0.2–1.5; *p* = 0.015) in males. In the MLR, leg strength was not associated with 6MWD.

### Asthma control and hospitalization

**FIGURE 2:**
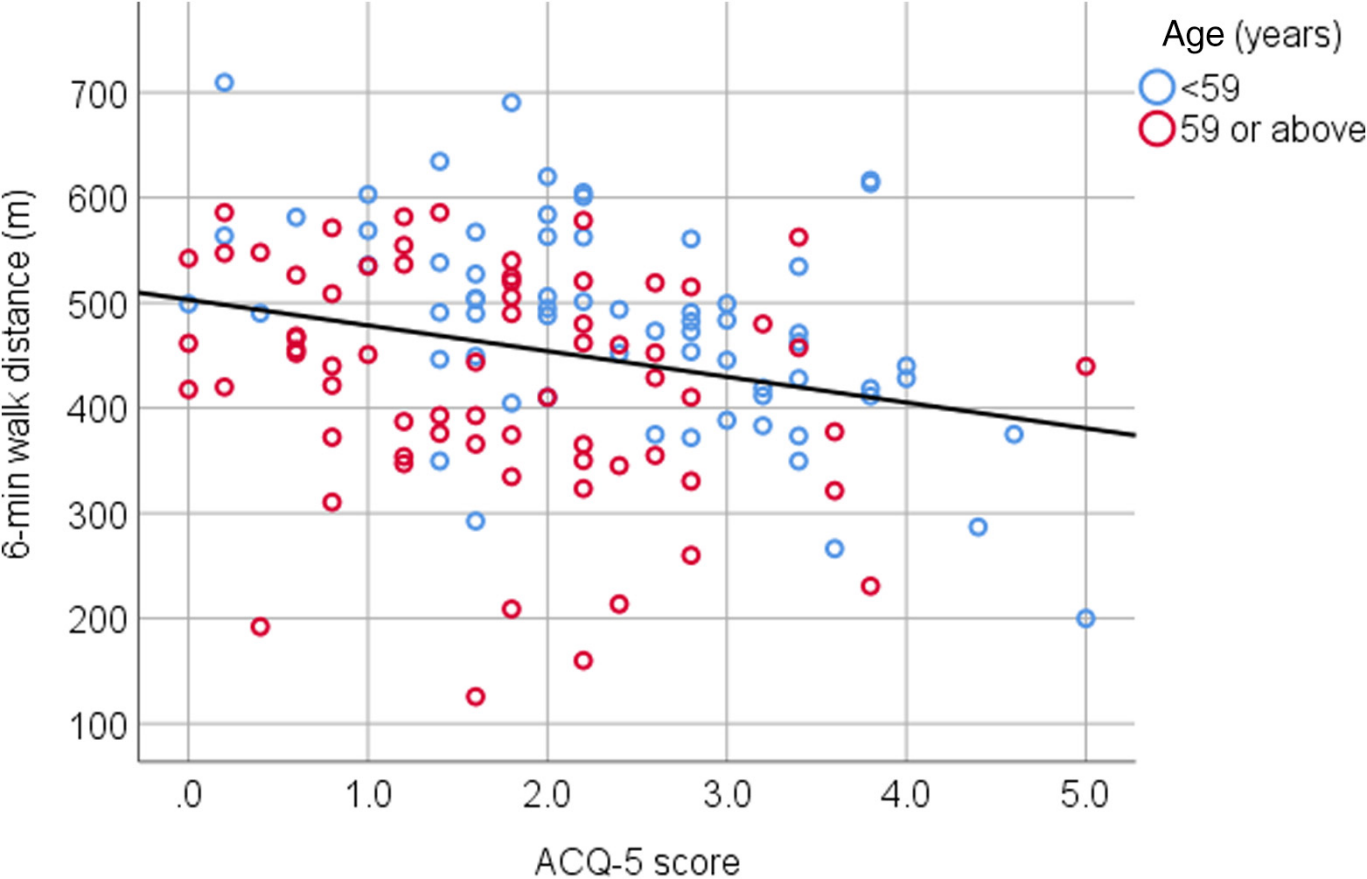
Relationship between 6‐min walk distance (6MWD) and ACQ‐5 (5‐item Asthma Control Questionnaire) score. Relationship between 6MWD and asthma control, both sexes included. Red circles indicate age above the median age (59 years), and blue circles indicate age below median age. Unadjusted, *r* = −0.259 (*p* = 0.002). When adjusted for age, asthma control score correlated inversely with a fair strength to 6MWD (*r* = −0.419, *p* < 0.0001).

There was a linear relationship between asthma control (ACQ‐5) and 6MWD (Figure 2). A 0.5‐point reduction in ACQ was associated with an increased walk distance of 10.2 m (95% CI −2.4 to 22.8) in males, 14.5 m (95% CI 4.5–24.5) in females and 15.2 m (95% CI 7.7–22.6) for the overall group. Exacerbations requiring hospitalization were not associated with 6MWD in the MLR analyses.

### Comorbidities

In the entire group and in males, depression was associated with a reduction in 6MWD by 46 m (95% CI 12–81; *p* = 0.009) and 62 m (95% CI −123 to 0; *p* = 0.048), respectively, using the SLR analysis (Table 2). No significant associations were seen between depression and anxiety in any MLR analyses (Tables 3 and 4). The significance of cardiac disease in a simple regression was >0.2 and thus not included in MLR analysis.

## DISCUSSION

We aimed to determine traits that impact exercise capacity in adults with severe asthma. We found that asthma symptoms (ACQ) and BMI were significantly correlated with exercise capacity in severe asthma. When analysed by sex, asthma control and BMI remained significantly related to 6MWD in females, but not in males. These results provide us with potential targets for optimizing exercise capacity and consequently gaining associated health benefits in patients with severe asthma.

The low percentage of predicted 6MWD indicates that the detrimental effects of severe asthma on exercise capacity impact both sexes, supporting the concept that exercise capacity may be a potential treatable target in both men and women. In females, an increase of one unit in BMI is associated with a reduction of 4.0 m in walk distance, corresponding to approximately 1% of the mean 6MWD. Data from the International Severe Asthma Registry (ISAR) estimate that seven of 10 people with severe asthma are either overweight (31%) or obese (39%).^27^ Furthermore, obesity is associated with negative outcomes in asthma.^28^ Given the frequency and magnitude of elevated BMI, both in our study and generally according to ISAR, we consider the association between BMI and 6MWD to be of clinical relevance. Combined with the detrimental effects of being overweight on asthma control, weight reduction should be a target for interventions to improve exercise capacity and asthma control, perhaps particularly in females. This is already widely acknowledged and several diet, exercise or combined interventions have been performed in overweight and obese individuals with asthma, but few in severe asthma.^3^, ^29^

The implications of elevated levels of serum IL‐6 on asthma control are not fully understood. The impact of exercise interventions on IL‐6 levels in asthma patients is inconsistent in randomized clinical trials.^30^, ^31^, ^32^ This lack of straight‐forward relationship probably reflects differences in baseline levels of included participants, as well as the fact that IL‐6 can be produced in response to exercise in skeletal muscle, and by adipose tissue.^33^ A cross‐sectional study by Peters et al., in asthma patients of various asthma severities, reported that elevated IL‐6 was significantly associated with a higher BMI, lower FEV1 and more frequent exacerbations.^34^ In our study, IL‐6 was significantly associated with 6MWD in females using the SLR, with an inverse relationship. However, the relationship diminished in the MLR, and the clinical importance seems to be negligible. With IL‐6 level being related to obesity, our data suggest that it is more appropriate to target BMI with weight reduction rather than IL‐6 itself, when aiming to improve exercise capacity in females.^35^, ^36^

The impact of leg strength on exercise capacity, and potential effects of improving leg strength, in adults with severe asthma are largely unknown. We found no association of leg strength with 6WMD in any of the MLR analyses. A meta‐analysis in patients with chronic obstructive pulmonary disease (COPD) showed no effect of adding resistance training to aerobic training in improving walk distance or maximal oxygen uptake.^37^ While there are differences in underlying pathophysiology, severe asthma and COPD share common traits, including impaired physical activity and detrimental impacts on body composition.^9^, ^10^, ^12^, ^38^, ^44^ Improving muscle strength is essential in treating patients with COPD, where skeletal muscle dysfunction is common and associated with mortality risk and impaired ability to handle daily activities.^39^ Several mechanisms responsible for muscle weakness are identified including chronic treatment with systemic glucocorticoids, which have also been demonstrated in patients with asthma.^39^, ^40^, ^41^ Add‐on resistance training of lower limbs in severe asthma could provide a sensible and feasible target, with a potential to improve asthma‐related QoL. However, it does not seem to affect 6MWD directly.

Surprisingly, we found no negative association between cardiac disease and walk distance, but this could be due to the limited cases of heart failure, and the self‐reported nature of the comorbidity.^42^ Anxiety and depression were common in our cohort and are generally frequent comorbidities in people with severe asthma.^43^ We found no association between depression or anxiety with 6MWD in the MLR analysis. However, in a feasibility study in severe asthma patients by Majd et al., anxiety and depression were predictors of non‐completion of the pulmonary rehabilitation intervention. The authors suggested addressing these extra‐pulmonary traits prior to exercise interventions.^18^

The association of asthma control on 6WMD seems clinically relevant. Interpretation of the association of asthma control is complex but could simply reflect that the perception of asthma symptoms impacts how much patients engage in physical activity and exercise and vice versa. The key clinical difference between the two sexes was the impact of BMI, which is also seen in healthy individuals.

There are some limitations to our study. Given the cross‐sectional design, causality cannot be inferred, and it is plausible that the relationship is bidirectional. We used the 6MWT, a submaximal exercise performance test, to assess exercise capacity as it is well validated in chronic respiratory diseases with moderate to very strong correlation to maximal exercise performance and physical activity level.^20^, ^21^ We did not address musculoskeletal conditions, that may impact exercise capacity. Refer Appendix S1 in the Supporting Information for self‐reported limitations to the 6MWT. Anxiety, depression and cardiac disease were self‐reported, and therefore some caution should be taken interpreting these results. For sensitivity analyses, we included all parameters in the MLR analyses (Appendix S1 and Tables S2–S4 in the Supporting Information).

In conclusion, we found that asthma symptoms and BMI in severe asthma were associated with exercise capacity. When split by sex, asthma symptoms and BMI were associated with exercise capacity in females, while no association was found in males. Leg strength and depression were not significantly associated to 6MWD in our study but may still be suitable targets for future treatment. Optimizing these factors could enhance the ability of patients to improve their exercise capacity and thereby gain the associated positive health outcomes. Studies evaluating a sequential approach optimizing of these factors, such as diet to reduce BMI and resistance training to improve leg strength, prior to aerobic exercise interventions versus simultaneous multidisciplinary interventions are needed.

## AUTHOR CONTRIBUTION

**Anders Pitzner‐Fabricius:** Conceptualization (equal); formal analysis (lead); methodology (equal); visualization (lead); writing – original draft (lead). **Vanessa L. Clark:** Conceptualization (supporting); data curation (supporting); formal analysis (equal); investigation (supporting); methodology (supporting); supervision (equal); validation (lead); writing – review and editing (supporting). **Vibeke Backer:** Conceptualization (equal); formal analysis (supporting); methodology (supporting); supervision (supporting); writing – review and editing (equal). **Peter G. Gibson:** Conceptualization (supporting); data curation (equal); funding acquisition (equal); investigation (equal); methodology (equal); resources (equal); writing – review and editing (equal). **Vanessa M. McDonald:** Conceptualization (equal); data curation (equal); formal analysis (equal); funding acquisition (equal); investigation (equal); methodology (equal); project administration (equal); resources (equal); supervision (lead); validation (equal); visualization (supporting); writing – review and editing (lead).

## CONFLICTS OF INTEREST

Vanessa M. McDonald was supported by an NHMRC TRIP fellowship; has participated in educational symposia funded by GlaxoSmithKline, AstraZeneca, Menarini and Novartis; and has participated in advisory boards for GlaxoSmithKline, AstraZeneca and Menarini. Peter G. Gibson holds an NHMRC Practitioner Fellowship; has participated in educational symposia funded by AstraZeneca, Boehringer Ingelheim, GlaxoSmithKline and Novartis; and has participated in studies funded by GlaxoSmithKline and AstraZeneca. Vibeke Backer reports grants and/or consulting and/or lecture fees from UNION therapeutics, Chiesi, Novartis, SanofiGenzyme, Pharmaxis, GlaxoSmithKline, Boehringer‐Ingelheim and TEVA; had travel expenses to congress meeting covered by SanofiGenzyme; is advisory board member at TEVA, Chiesi, AstraZeneca, SanofiGenzyme, Novartis and ALK‐Abello; and has received study medication from Novartis. Vanessa L. Clark receives a fellowship from the National Health and Medical Research Council Centre of Research Excellence in Severe Asthma. Anders Pitzner‐Fabricius has no conflicts of interest.

## HUMAN ETHICS APPROVAL DECLARATION

Ethics approval was granted by the human research ethics committees of the Hunter New England Local Health District (08/08/20/3.10), Australia. The study was conducted according to Good Clinical Practice Guidelines and each participant provided written informed consent.

## Supporting information


**Supporting information**.

## Data Availability

The data that support the findings of this study are available from the corresponding author upon reasonable request.
