# Supplementary material for: Factors associated with 6‐min walk distance in severe asthma: A cross‐sectional study
Source: Respirology. 2022 Jul 10;27(12):1025–33. doi: 10.1111/resp.14323 (PMC9796104; doi:10.1111/resp.14323)
Supplement: Supplementary file 1 — Supporting information. [file RESP-27-1025-s001.docx]

Supporting Information

Factors associated with 6-minute walk distance in severe asthma: A cross-sectional study

A. Pitzner-Fabricius, VL. Clark, V. Backer, PG. Gibson, VM. McDonald

**Appendix S1-ADDITIONAL INFORMATION**

**Procedures**

The Asthma Quality of Life Questionnaire (AQLQ) is a disease specific quality of life tool that measures the functional impairments that most concern people with asthma. The questionnaire consists of 32 questions, a seven-point Likert scale of responses is provided for each question. The questionnaire assesses the impact of asthma on quality of life over the previous 2 weeks. The four domains covered include; asthma symptoms (12 items), activity limitation (11 items), emotional function (5 items) and environmental stimuli (4 items). An improvement of 0.5 or greater is considered the minimal clinically important difference (MCID).^1^

Fraction of exhaled nitric oxide was measured by a single breath test using a chemiluminescence Fractional exhaled nitric oxide (FeNO) (ANALYZER CLD 88 Series with DENOX 88, Duernten, Switzerland). Tests were carried out in accordance with ERS/ATS guidelines with an expiratory flow of 50 ml/s.^2^

**Self-reported limitations to the 6 Minute Walk Test**

At completion of the 6 MWT participants were asked about limitations to their test.

- 8 of 137 reported shortness of breath or chest tightness as exercise test limitation during the 6MWT.
- 33 reported some pain in the lower extremities/hip as some sort of limitation to the exercise test.

**References**

1. Juniper EF, Guyatt GH, Ferrie PJ, Griffith LE. Measuring Quality of Life in Asthma. *Am Rev Respir Dis*. 1993;147(4):832-838. doi:10.1164/ajrccm/147.4.832

2. Measurement O. ATS/ERS recommendations for standardized procedures for the online and offline measurement of exhaled lower respiratory nitric oxide and nasal nitric oxide, 2005. *Am J Respir Crit Care Med*. 2005;171(8):912-930. doi:10.1164/rccm.200406-710ST

**Table S1.** Association of biological and clinical factors with 6MWD in both sexes using a multiple linear regression model adjusted for age.

| 6MWD | **Multiple linear regression analysis both sexes** | | | |
| --- | --- | --- | --- | --- |
|  | Mean change (95% CI) | β | Significance | adj. *R*^2^ |
| ACQ-5 | -15.2 (-22.6 to -7.7) | -.381 | **.0001** | .425 |
| BMI | -3.2 (-5.1 to -1.3) | -.266 | **.001** |  |
| Prebronchodilator FEV1 % predicted | -0.1 (-0.8 to 0.6) | -.019 | .816 |  |
| Hospitalisation | -26 (-60 to 7) | -.123 | .121 |  |
| IL-6, pg/ml | -3.0 (-6.1 to 0.1) | -.151 | **.**058 |  |
| Isometric leg strength | 0.3 (-0.2 to 0.7) | .133 | .237 |  |
| Anxiety | -20 (-55 to 15) | -.089 | .258 |  |
| Depression | 13 (-20 to 47) | .072 | .436 |  |
| Age | -3.1 (-4.2 to -2.0) | -.505 | **<.001** |  |
| Sex | 5 (-36 to 45) | .024 | .824 |  |

*6MWD,* 6-minute walk distance; *ACQ-5*, 5-item Asthma Control Questionnaire; *BMI*, body mass index; *IL*, interleukin; *FEV1*, forced expiration in 1 second.

Results are presented as change in 6MWD (in metres) per 1-unit change (0.5 for ACQ-5) in the biological or clinical factor, and as a regression coefficient (β). Bold p value denotes statistical significance.

**Table S2** Association of biological and clinical factors with 6MWD in both sexes using a multiple linear regression model, with all biological and clinical parameters included, adjusted for age.

| 6MWD | **Multiple linear regression analysis both sexes** | | | |
| --- | --- | --- | --- | --- |
|  | Mean change (95% CI) | β | Significance | adj. *R*^2^ |
| ACQ-5 | -15.0 (-22.5 to -7.5) | -.377 | **.0001** | .420 |
| BMI | -3.1 (-5.0 to -1.2) | -.258 | **.002** |  |
| Prebronchodilator FEV1 % predicted | -0.1 (-0.8 to 0.6) | -.024 | .771 |  |
| Hospitalisation | -27 (-60 to 7) | -.123 | .120 |  |
| IL-6, pg/ml | -3.1 (-6.2 to 0.1) | -.154 | .056 |  |
| Isometric leg strength | 0.3 (-0.2 to 0.7) | .127 | .265 |  |
| Cardiac disease | -9 (-44 to 25) | -.045 | .595 |  |
| Anxiety | -19 (-54 to 16) | -.086 | .278 |  |
| Depression | 14 (-20 to 48) | .076 | .413 |  |
| Age | -3.0 (-4.2 to -1.9) | -.491 | **<**.**00001** |  |
| Sex | 4 (-37 to 44) | .019 | .862 |  |

*6MWD,* 6-minute walk distance; *ACQ-5*, 5-item Asthma Control Questionnaire; *BMI*, body mass index; *FEV1*, forced expiration in 1 second; *IL*, interleukin.

Results are presented as change in 6MWD (in metres) per 1-unit change (0.5 for ACQ-5) in the biological or clinical factor, and as a regression coefficient (β). Bold p value denotes statistical significance.

**Table S3-** Association of biological and clinical factors with 6MWD in females using a multiple linear regression model, with all biological and clinical parameters included, adjusted for age.

| 6MWD | Multiple linear regression analysis in females | | | |
| --- | --- | --- | --- | --- |
|  | Mean change (95% CI) | β | Significance | adj. *R*^2^ |
| ACQ-5 | -12.2 (-21.1 to -3.3) | -.320 | **.008** | .551 |
| BMI | -3.8 (-5.7 to -1.9) | -.388 | **.0002** |  |
| Prebronchodilator FEV1 % predicted | -0.2 (-1.1 to 0.7) | -.043 | .660 |  |
| Hospitalisation | -31 (-66 to 4) | -.162 | .082 |  |
| IL-6, pg/ml | -5.2 (-8.2 to -2.3) | -.321 | **.001** |  |
| Isometric leg strength | -0.2 (-0.8 to 0.4) | -.064 | .523 |  |
| Cardiac disease | -29 (-67 to 9) | -.152 | .130 |  |
| Anxiety | -9 (-45 to 26) | -.045 | .608 |  |
| Depression | 28 (-5 to 62) | .170 | .097 |  |
| Age | -2.5 (-3.8 to -1.2) | -.449 | **.0002** |  |

*6MWD,* 6-minute walk distance; *ACQ-5*, 5-item Asthma Control Questionnaire; *BMI*, body mass index; *IL*, interleukin; *FEV1*, forced expiration in 1 second.

Results are presented as change in 6MWD (in metres) per 1-unit change (0.5 for ACQ-5) in the biological or clinical factor, and as a regression coefficient (β). Bold p value denotes statistical significance.

**Table S4.** Multiple linear regression analysis in males, with all biological and clinical parameters included, adjusted for age.

| 6MWD | Multiple linear regression analysis in males | | | |
| --- | --- | --- | --- | --- |
|  | Mean change (95% CI) | β | Significance | adj. *R*^2^ |
| ACQ-5 | -16.4 (-33.6 to 0.9) | -.374 | .062 | .265 |
| BMI | -1.5 (-7.4 to 4.5) | -.079 | .620 |  |
| Prebronchodilator FEV1 % predicted | -0.0 (-1.6 to 1.5) | -.010 | .953 |  |
| Hospitalisation | -50 (-141 to 41) | -.194 | .272 |  |
| IL-6, pg/ml | 3.5 (-7.5 to 14.4) | .114 | .522 |  |
| Isometric leg strength | 0.6 (-0.3 to 1.4) | .237 | .175 |  |
| Cardiac disease | -0 (-72 to 72) | .000 | 1.000 |  |
| Anxiety | -41 (-125 to 42) | -.161 | .317 |  |
| Depression | -3 (-99 to 92) | -.014 | .943 |  |
| Age | -3.4 (-6-0 to -0.9) | -.462 | **.011** |  |

*6MWD,* 6-minute walk distance; *ACQ-5*, 5-item Asthma Control Questionnaire; *BMI*, body mass index; *IL*, interleukin; *FEV1*, forced expiration in 1 second.

Results are presented as change in 6MWD (in metres) per 1-unit change (0.5 for ACQ-5) in the biological or clinical factor, and as a regression coefficient (β). Bold p value denotes statistical significance.
